# Relationship between diet, sociodemographic factors, and body composition in students from UNEMI and ESPOCH

**DOI:** 10.3389/fpubh.2025.1621661

**Published:** 2025-07-24

**Authors:** Katherine Denisse Suaárez-González, Angélica María Solis-Manzano, María Victoria Padilla-Samaniego, Verónica Patricia Sandoval-Tamayo, Edgar Rolando Morales-Caluña

**Affiliations:** State Universitty of Milagro, Faculty of Health Sciences, Milagro, Ecuador

**Keywords:** university diet, body composition, sociodemographic factors, cardiovascular health, Ecuadorian students

## Abstract

**Introduction:**

The nutritional habits and sociodemographic background of university students are critical factors influencing their body composition and long-term health. This study aimed to analyze the relationship between diet quality, sociodemographic factors, and body structure among students from the State University of Milagro (UNEMI) and the Higher Polytechnic School of Chimborazo (ESPOCH) in Ecuador.

**Methods:**

A cross-sectional design was applied to a sample of 404 students selected through stratified random sampling. Data were collected via structured interviews, 24-hour dietary recall, and food frequency questionnaires, along with anthropometric and clinical evaluations. Statistical analyses included one-way ANOVA and Kruskal–Wallis tests to assess associations, stratified by sex.

**Results:**

Only 2% of students maintained a nutrient-rich diet, while over 78% had nutrient-poor dietary patterns. Among women, higher fat intake was significantly associated with elevated triglyceride levels (*p* = 0.010), and nutrient-rich diets correlated with lower systolic blood pressure (*p* = 0.000). Among men, high-fat diets were linked to increased systolic and diastolic blood pressure (*p* = 0.007 and *p* = 0.004, respectively). Sociodemographic analysis revealed that men born in the Highland region had significantly higher body fat percentage and hip circumference than those from the Coastal region. A trend in muscle mass percentage according to paternal origin was also observed.

**Conclusion:**

The findings highlight the importance of addressing both dietary behaviors and sociodemographic contexts in university health promotion. Nutritional education tailored to regional and cultural backgrounds, alongside strategies to promote physical activity, could support healthier body composition and reduce cardiovascular risks in young adults.

## 1 Introduction

Body composition results from a complex web of factors, including intrinsic biological elements, such as genetic mechanisms that may cause imbalances (1), as well as modifiable lifestyle factors like physical activity (2) and dietary patterns, encompassing both meal timing and circadian rhythm disturbances (3).

Several studies have shown that sociodemographic variables such as age, sex, and socioeconomic status play a crucial role in the quality of diet, significantly impacting body composition (4). Within the university context, students with higher educational attainment tend to adopt healthier eating patterns, resulting in better diet quality and consequently more favorable body composition (5). However, multiple factors such as lack of time, irregular physical activity (6), and economic constraints can deteriorate diet quality among university students. Gender differences have also been reported, with men generally showing less healthy dietary practices (7).

Students’ nutrition is not solely determined by individual choices or food availability but is deeply influenced by the academic environment. Stress arising from academic responsibilities can significantly alter dietary patterns, adversely affecting physical wellbeing (8, 9), promoting sedentary lifestyles (10), and encouraging harmful eating behaviors, thereby increasing the risk of hypertension and obesity (11). Recent studies have found a significant relationship between the consumption of high-calorie density foods and increased visceral fat and body weight, highlighting the importance of adequate dietary quality for cardiovascular health (12, 13).

Additionally, nutritional status differences and weight variations among university students, particularly health sciences students, reflect not only individual habits but also the interaction of socioeconomic and cultural factors. Research by Cheli Vettori et al. (14) emphasizes the economic context’s influence on food choices, directly impacting cardiovascular health and overall wellbeing. This dynamic is exacerbated by inequalities in access to healthy food and physical activity opportunities, strongly tied to students’ socioeconomic levels. Furthermore, academic stress has been noted to exacerbate these effects, promoting sedentary lifestyles and poor dietary choices (10).

In this context, the central aim of this study is to analyze the influence of diet and sociodemographic factors on the structure and body composition of students from the State University of Milagro (UNEMI) and the Higher Polytechnic School of Chimborazo (ESPOCH), providing evidence to support the design of nutritional intervention strategies tailored to this population.

## 2 Materials and methods

### 2.1 Sample selection, inclusion and exclusion criteria

A total of 404 university students from the State University of Milagro (UNEMI) and the Higher Polytechnic School of Chimborazo (ESPOCH) were selected using a stratified random sampling method. Inclusion criteria included students aged between 18 and 55 years, currently enrolled in undergraduate programs at the time of data collection, who voluntarily agreed to participate in the study. This broad age range reflects the demographic reality of Ecuadorian public universities, where many students are non-traditional and return to formal education later in life. Exclusion criteria included individuals with a prior diagnosis of cardiovascular disease or a chronic illness that could affect cardiovascular biomarkers, as well as those who did not complete the dietary or clinical assessments.

### 2.2 Study context

The study involved students from two distinct regions of Ecuador: The Coastal and Andean Highland areas. Participants from UNEMI were predominantly from Milagro, a mid-sized city located in the Coastal region, characterized by a warm tropical climate, low-altitude geography, and an economy based on agriculture, agro-industry, and commerce. In contrast, students from ESPOCH were mainly from Riobamba and surrounding areas in the Andean Highlands, located at a much higher altitude with a temperate to cold climate. This region’s economy is more rural, based on agriculture, small-scale trade, and public services, facing geographic and economic barriers to healthcare and nutritional resources.

Income level classifications were operationalized based on regional economic activity, public infrastructure access, and degree of urbanization. The Coastal region, especially Guayaquil and neighboring cities like Milagro, was categorized as a higher-income area due to greater industrial and commercial activity, better healthcare services, and connectivity. The Highland region, particularly rural areas near Riobamba, was classified as a lower-income area characterized by lower family income averages, limited specialized medical care access, and greater prevalence of food insecurity and poverty. These contextual differences could partly explain disparities observed in cardiovascular health indicators between the two student populations.

### 2.3 Sample size calculation

The sample size was calculated using a 95% confidence level, a 5% margin of error, and an estimated population of approximately 8,000 students from both universities. Based on these parameters, the minimum required sample size was 367 students. A total of 404 students participated, ensuring representativeness and accounting for possible non-responses or data inconsistencies.

### 2.4 Ethical considerations and data protection

This study was conducted in accordance with the Declaration of Helsinki. All procedures involving human participants were approved by the Human Research Ethics Committee of ESPOCH (approval number IO-07-CEISH-ESPOCH-2023). Written informed consent was obtained from all participants. Data confidentiality was guaranteed by anonymizing the information and securely storing it in encrypted databases accessible only to the research team.

### 2.5 Data collection procedures

Sociodemographic and dietary data were obtained through structured interviews. Clinical data were collected by qualified personnel in university health centers. Anthropometric and biochemical evaluations included blood pressure measurements, oxygen saturation, and blood analyses for lipid profiles, glycemia, and cholesterol levels.

### 2.6 Dietary assessment

Dietary intake was assessed using a 24-h recall and a food frequency questionnaire adapted from the Block Screening Questionnaire (15). Macronutrient and micronutrient intake (iron, calcium, vitamin C) were estimated using regional food composition tables from FAO/INFOODS.

### 2.7 Diet classification

Fat intake was categorized as very high (≥50 points), high (40–49), moderate (30–39), low (20–29), and very low (<20).

Nutritional adequacy was classified into three levels:

Nutrient-rich diets: over 80% of daily recommendations for at least three key nutrients.

Sufficiently nutrient diets: between 50 and 80% of recommendations.

Nutrient-poor diets: less than 50% of recommended intake.

### 2.8 Data analysis and statistical tests

Descriptive statistics were used to summarize demographic variables, dietary patterns, and body composition. One-way analysis of variance (ANOVA) was applied to evaluate differences in body health indicators across diet and sociodemographic groups. Normality was tested using the Shapiro–Wilk test. When assumptions of normality or homoscedasticity were violated, non-parametric tests (Kruskal-Wallis) were considered. Post-hoc comparisons were adjusted using the Bonferroni correction when necessary.

### 2.9 Association measures and confounder adjustments

Effect sizes (Eta squared – η^2^) were calculated to evaluate the magnitude of observed differences in ANOVA tests. Analyses were stratified by sex to account for physiological differences. Although no multivariable regression models were applied, the potential influence of confounding variables is acknowledged as a study limitation.

### 2.10 Body composition and structure

Anthropometric assessment included measurements of: body weight, skinfold thickness (triceps, subscapular, biceps, suprailiac), body circumferences (relaxed arm, waist, hip), and bone diameters (transverse thorax, anteroposterior thorax, biacromial diameter). Total body fat percentage and total muscle mass percentage were measured via bioelectrical impedance analysis, following standardized techniques per ISAK protocol (16).

### 2.11 Dietary characteristics

Food consumption was assessed using a quantitative method (24-h recall) and a qualitative method (specific food frequency questionnaire). Percentages of adequacy for energy, macronutrients, and micronutrients (iron, calcium, vitamin C) were calculated, along with consumption frequencies.

## 3 Results

### 3.1 Statistical characteristics of the sample

The sample consisted of 404 university students, with a gender distribution of 41.67% men and 58.33% women. The mean age was 23.34 years (SD = 4.38), with a median of 22 years and an age range from 19 to 55 years.

Regarding dietary fat intake, 22% of participants had a very high fat intake, 7% had a high intake, 16% had a moderate intake, 26% had a low intake, and 29% followed an almost fat-free diet. Concerning fruit, vegetable, and fiber consumption, only 2% maintained a nutrient-rich diet, while over 78% maintained a nutrient-poor diet (see Table 1).

**Table 1:** Sociodemographic characteristics of the participants.

Variable	Total (*n* = 404)	Men (*n* = 168)	Women (*n* = 236)
Age (mean ± SD)	23.34 ± 4.40	23.70 ± 4.50	23.10 ± 4.30
Age range	19–55	–	–
Place of birth			
Place of birth–coastal region	180 (44.60%)	80 (47.60%)	100 (42.40%)
Place of birth–highland region	200 (49.50%)	78 (46.40%)	122 (51.70%)
Place of birth–other	24 (5.90%)	10 (6%)	14 (5.90%)
Subtotal (%)	100%	100%	100%
Current residence			
Current residence–Milagro	220 (54.50%)	100 (59.50%)	120 (50.80%)
Current residence–Riobamba	160 (39.60%)	60 (35.70%)	100 (42.40%)
Current residence–other	24 (5.90%)	8 (4.80%)	16 (6.80%)
Subtotal (%)	100%	100%	100%
Father’s place of origin			
Father’s place of origin–coastal region	190 (47.03%)	90 (53.60%)	100 (42.40%)
Father’s place of origin–highland region	190 (47.03%)	70 (41.70%)	120 (50.80%)
Father’s place of origin–other	24 (5.94%)	8 (4.80%)	16 (6.80%)
Subtotal (%)	100%	100%	100%
Mother’s place of origin			
Mother’s place of origin–coastal region	200 (49.50%)	85 (50.60%)	115 (48.70%)
Mother’s place of origin–highland region	180 (44.60%)	75 (44.60%)	105 (44.50%)
Mother’s place of origin–other	24 (5.90%)	8 (4.80%)	16 (6.80%)
Subtotal (%)	100%	100%	100%

Percentages are calculated vertically by column for each sex group to facilitate comparison across sociodemographic categories. Totals do not sum to 100% when read by row, as this table is intended to show distribution within each sex.

Variables such as place of birth, current residence, and parental place of origin were distributed between the Coastal and Highland regions, with slight differences between men and women.

### 3.2 Diet and body composition indicators

Table 2 summarizes the results of the one-way ANOVA assessing the relationship between dietary factors (fat intake and fruit, vegetable, and fiber scores) and cardiovascular health indicators. Among women, significant differences in triglyceride levels were observed based on fat intake [ANOVA, *F*(4, 230) = 3.50, *p* = 0.010]. Women with a very high-fat diet had a mean triglyceride level of 189.6 mg/dL (SD = 35.20), compared to 132.40 mg/dL (SD = 28.70) in those with a low-fat diet.

**Table 2:** Results of the one-way analysis of variance between diet and body composition indicators (*p* < 0.09).

Group	Indicator	Mean (SD)	*F*-statistic	*p*-value
Women–very high fat intake	Triglycerides (mg/dL)	189.6 (35.2)	3.50	0.010
Women–low fat intake	Triglycerides (mg/dL)	132.4 (28.7)		
Men–high fat intake	Systolic BP (mmHg)	128.3 (9.2)	3.80	0.007
Men–low fat intake	Systolic BP (mmHg)	117.4 (7.8)		
Men–very high fat intake	Diastolic BP (mmHg)	83.7 (8.6)	4.17	0.004
Men–low fat intake	Diastolic BP (mmHg)	76.2 (7.4)		
Women–nutrient-rich diet	Systolic BP (mmHg)	106.5 (7.1)	19.30	0.000
Women–needs supplementation	Systolic BP (mmHg)	113.7 (9.3)		

This table includes only those comparisons where statistically significant differences were observed (*p* < 0.05). Non-significant variables were excluded for clarity. BP: blood pressure.

In men, systolic blood pressure was significantly higher among those consuming a high-fat diet (mean = 128.30 mmHg, SD = 9.20) compared to those on a low-fat diet (mean = 117.40 mmHg, SD = 7.80) [*F*(4, 164) = 3.80, *p* = 0.007]. Diastolic blood pressure also showed significant differences [*F*(4, 164) = 4.17, *p* = 0.004], being higher in those with a very high-fat diet (mean = 83.70 mmHg, SD = 8.60) compared to those with a low-fat diet (mean = 76.20 mmHg, SD = 7.40). Among women, those with a nutrient-rich diet exhibited lower systolic blood pressure (mean = 106.50 mmHg, SD = 7.10) than those needing dietary supplementation (mean = 113.70 mmHg, SD = 9.30) [*F*(2, 23) = 19.30, *p* = 0.000].

### 3.3 Sociodemographic factors and body composition indicators

The one-way ANOVA analysis showed that place of birth significantly influenced body fat percentage among men. Men born in the Highlands had an average body fat percentage of 21.4% (SD = 4.9), compared to 19.8% (SD = 4.3) for those born in the Coastal region (*F* = 3.854, *p* = 0.026).

Significant differences in hip circumference were also observed: men from the Highlands had an average hip circumference of 97.5 cm (SD = 7.2) compared to 94.3 cm (SD = 6.8) among those from the Coastal region (*F* = 4.873, *p* = 0.011). Among women, waist circumference showed slight differences according to region of birth, but these were not statistically significant (*F* = 1.623, *p* = 0.202).

**Figure 1:**
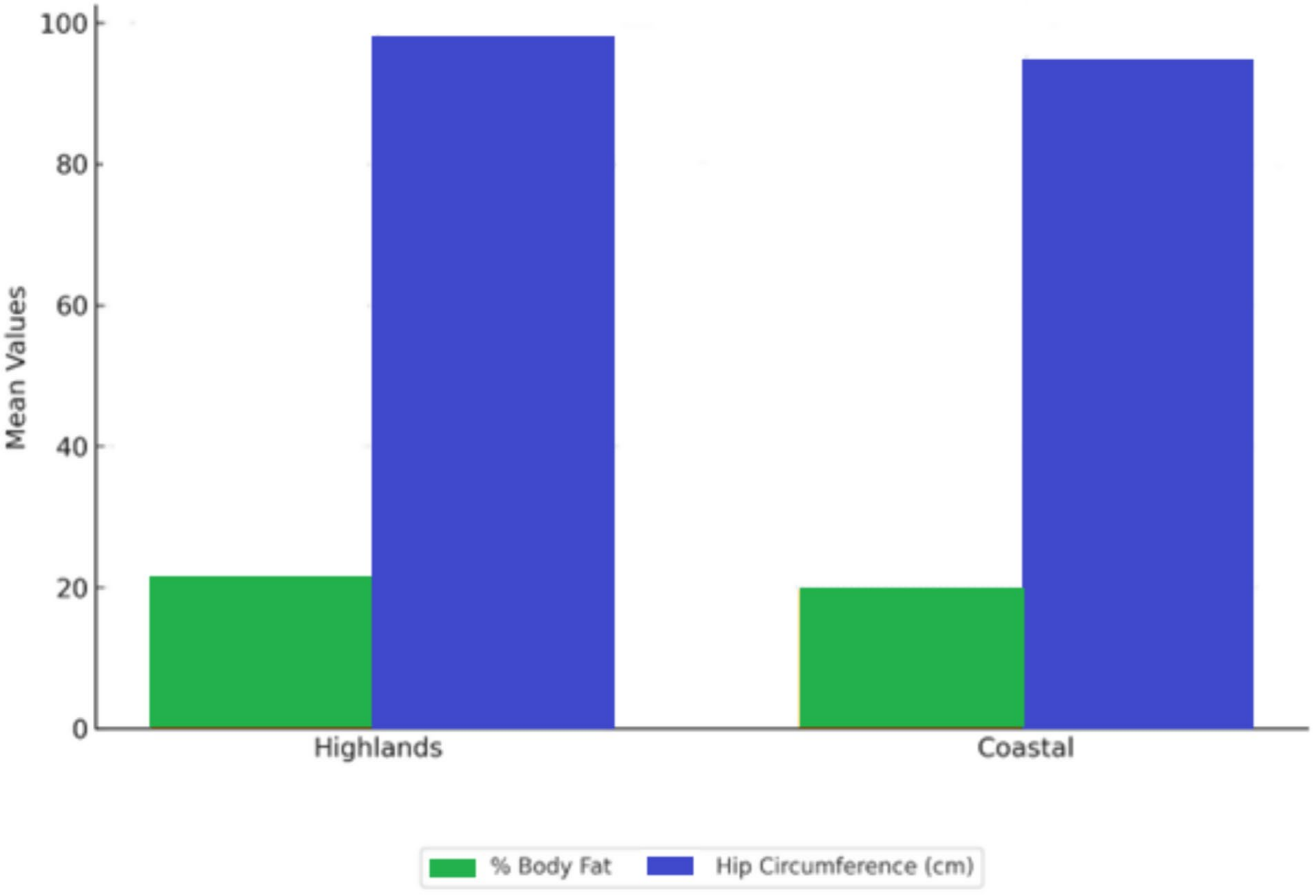
Body composition by birthplace.

These results are visually summarized in Figure 1, which highlights the regional differences in male body composition indicators.

### 3.4 Parental background and cardiovascular health

The place of origin of the father was associated with differences in muscle mass percentage among men. Participants whose fathers were from the Highlands showed an average muscle mass percentage of 43.2% (SD = 5.0), while those whose fathers were from the Coastal region had 44.9% (SD = 4.7) (*F* = 2.518, *p* = 0.088). Although this difference was not statistically significant at the 5% level, it suggests a trend indicating a potential influence of paternal origin on students’ body composition.

**Figure 2:**
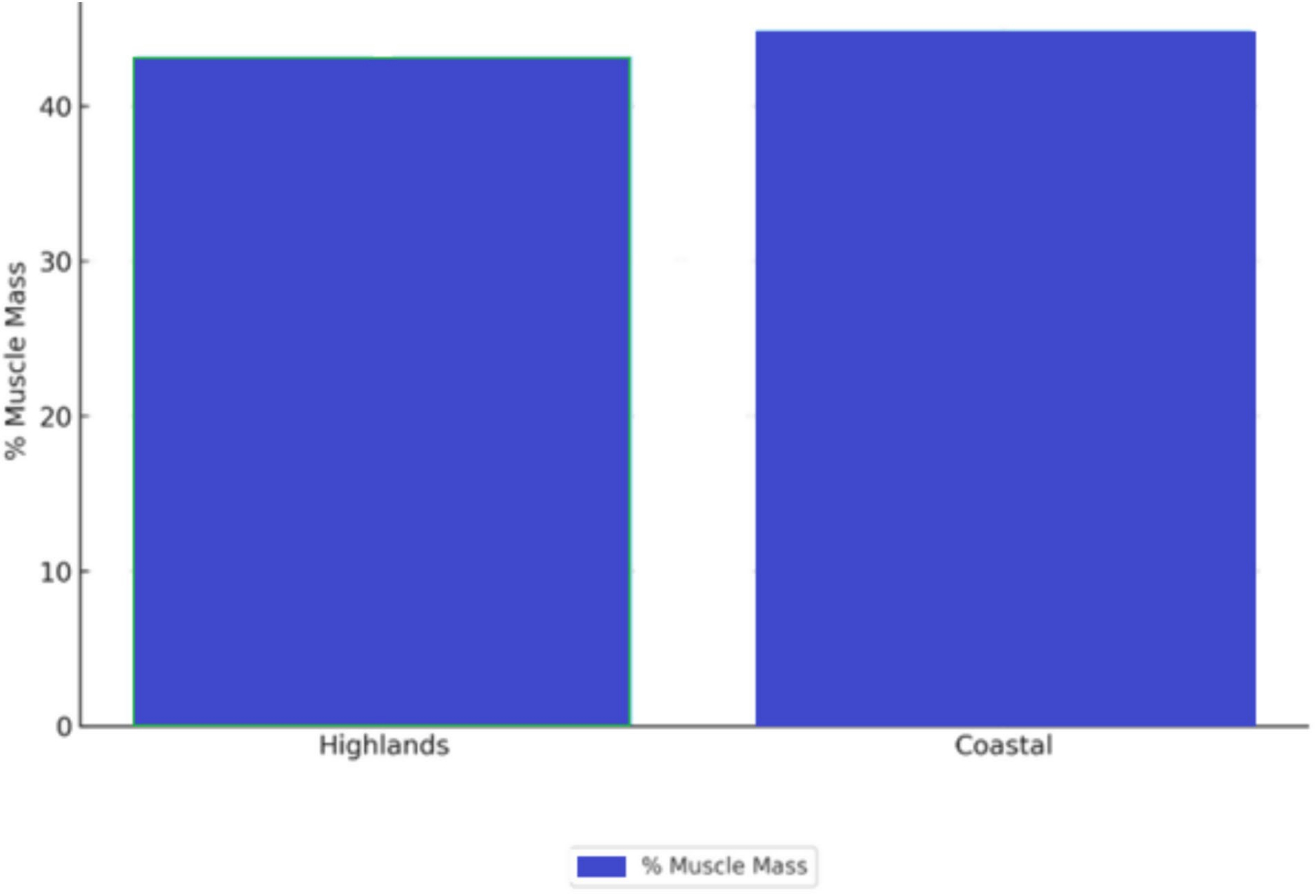
Muscle mass by paternal origin.

These trends are illustrated in Figure 2, which compares muscle mass percentages among male students according to their paternal region of origin.

## 4 Discussion

This study aimed to identify the influence of diet and sociodemographic factors on the structure and body composition of university students from public higher education institutions in Ecuador. The findings showed that only 2% of participants maintained a nutrient-rich diet and 29% reported an almost fat-free diet, a nutritional pattern similar to that observed among students at the University of Valladolid in Spain (17).

Regarding food intake and nutrients, no significant differences were found between men and women in the main body composition indicators. This finding contrasts with the study by Aparicio et al. (18), conducted with Mexican medical students, where a positive association between total energy consumption and muscle mass was observed. However, our analysis revealed that lower fat intake might be associated with a smaller arm circumference in women, suggesting a possible influence of diet quality on muscle mass development, in line with the findings of Pico Fonseca et al. (13), who documented the relationship between high-calorie density diets and increased visceral fat.

Similar findings were reported by Hernández-Calderón et al. (19) in a study of university students from Morelia, Mexico. They found notable anthropometric differences between sexes, with women showing lower muscle mass and higher fat percentage compared to men, as well as a prevalence of poor eating habits, particularly low intake of fruits and vegetables. These patterns are consistent with our observations and highlight the need for targeted nutritional strategies in university populations across Latin America.

Additionally, research by de Almeida et al. (12) highlights the connection between overweight, obesity, and irregular physical activity patterns, emphasizing the importance of maintaining proper nutrition and an active lifestyle to preserve body and metabolic health among university students.

Regarding sociodemographic factors, our study demonstrated that birthplace significantly influenced certain body composition indicators in men, such as body fat percentage and hip circumference. These findings could be explained by variations in dietary traditions and access to healthy foods across regions, supporting previous arguments about how environmental factors can shape eating habits and body morphology (8). This result contrasts with findings by Nieczuja-Dwojacka et al. (20), who found no morphological differences between rural and urban children, suggesting that regional influence may intensify with age.

In terms of parental influence, no significant associations were observed between maternal or paternal place of origin and body composition indicators among female university students. This finding aligns with studies such as those by Mesa et al. (21), who found no important effects of parental geographic origin on morphology in child populations. However, other factors, such as socioeconomic status and access to healthcare services, could have a more relevant mediating role in Ecuador’s transitional nutritional context (22).

It is also important to consider that the absence of statistically significant associations between parental origin and body composition—particularly muscle mass in men—may be influenced by unadjusted confounding variables such as age and physical activity. Although a trend was observed in the data, the lack of multivariable adjustment limits our ability to draw stronger conclusions. Future studies using generalized linear models or multivariate regression techniques that incorporate covariates such as age, physical activity level, and socioeconomic status may uncover significant relationships that remain hidden in the current univariate analysis.

Furthermore, consistent with observations by Cruz et al. (10) and Gasparotto et al. (11), it is noted that academic stress could indirectly contribute to suboptimal dietary patterns, encouraging sedentary lifestyles and resulting in alterations in body composition, although this effect was not directly measured in our study.

These results underscore the need for the implementation of specific nutritional intervention strategies for university students, adapted to their sociocultural contexts and life experiences. Integrating nutritional education programs into the university setting could positively impact the promotion of healthy eating habits and the prevention of body composition alterations (23).

Finally, practices such as mindful eating have proven effective in improving control over eating patterns and reducing emotional eating behaviors (24), representing an additional promising strategy for nutritional intervention programs targeting students.

Overall, the findings reaffirm the critical importance of promoting healthy lifestyles among university students, considering both dietary and sociodemographic influences to improve body health and prevent chronic diseases early in adulthood.

### 4.1 Limitations

This study has some limitations. First, the cross-sectional design prevents establishing causal relationships between dietary, sociodemographic factors, and body composition. Additionally, dietary assessment based on 24-h recall and food frequency questionnaires may be subject to memory bias and underreporting, common in self-reported studies. A key methodological limitation is the exclusive use of one-way ANOVA for statistical analysis. While this method allowed for initial group comparisons, it did not account for potential confounding variables or interactions among multiple predictors. Future research should apply multivariate approaches such as generalized linear models (GLMs), ANCOVA, or multiple regression to provide a more robust and comprehensive interpretation of the data.

Another limitation is the wide age range of participants (18–55 years), which, while reflective of the diverse student population in public universities, may introduce physiological variability that was not fully controlled for in the current analysis. Although the dataset was stratified by sex, future analyses should include age-adjusted models or limit the age range to reduce heterogeneity.

Additionally, the analysis of associations between parental background and body composition—particularly muscle mass in male participants—was limited by the absence of adjustments for confounding variables such as age and physical activity. Although trends were observed, the use of univariate methods may have obscured potentially significant relationships. More rigorous statistical approaches that control for these covariates are necessary to better understand the role of parental origin in shaping body composition.

### 4.2 Future research

Although this study provides valuable evidence on the influence of diet and sociodemographic factors on body composition among university students, several areas for future research are identified. First, conducting longitudinal studies would allow for analyzing dietary and body composition changes over time, establishing stronger causal relationships. In addition, future analyses should employ multivariable statistical models that adjust for key confounding variables such as age, socioeconomic status, physical activity, academic stress, and sleep patterns, to enhance analytical rigor.

We also recommend exploring narrower age bands in future data collection efforts, such as focusing on students aged 20–30 years, which would reduce variability and allow for more homogeneous subgroup analyses. Stratifying or adjusting by age in multivariable models would improve interpretation and better isolate the effects of dietary and demographic variables on body composition. A particularly relevant avenue for future research involves re-examining the influence of parental background—especially paternal origin—on muscle mass in male students. Although only trends were observed in this study, applying generalized linear models or multivariate regression techniques adjusted for age and physical activity could reveal significant associations that were not detected using univariate methods. This approach would provide greater insight into the intergenerational and sociocultural factors influencing body composition.

Furthermore, including other biomarkers (e.g., insulin levels, inflammatory markers) would expand the scope of metabolic health assessment in this population.

### 4.3 Practical implications

The findings of this study have important practical implications for the design and implementation of health promotion strategies in the university setting. First, the need to implement nutritional education programs adapted to students’ sociocultural context is emphasized, highlighting the importance of a balanced diet rich in fruits, vegetables, fiber, and healthy fats.

Considering the association found between dietary patterns and cardiovascular health indicators, awareness campaigns on the risks associated with excessive fat intake and poor diet quality are suggested, integrating educational activities, practical workshops, and individualized nutritional counseling.

Promoting active lifestyles through both curricular and extracurricular physical activity programs should also be prioritized to counteract the negative effects of sedentary behaviors observed among university students. Similarly, it is recommended to incorporate psychological support services and stress management programs as part of student wellness policies, given the potential impact of academic stress on eating habits and body composition.

Finally, universities could establish partnerships with local health services and community organizations to facilitate access to healthy foods and health promotion programs, thus contributing to the prevention of chronic diseases from early adulthood.

## 5 Conclusion

This study identified the influence of diet and sociodemographic factors on the structure and body composition of university students from the State University of Milagro (UNEMI) and the Higher Polytechnic School of Chimborazo (ESPOCH). The results showed that a small proportion of participants maintained nutritionally rich diets, while the majority exhibited suboptimal dietary patterns, characterized by low fruit, vegetable, and fiber intake and high fat consumption.

Although no statistically significant differences were found in body composition indicators between men and women according to diet quality, relevant trends were observed, such as a smaller arm circumference in women with lower fat intake, suggesting a potential impact of diet quality on muscle mass development. Additionally, the relationship between high-fat intake and cardiovascular risk parameters, such as elevated triglyceride levels and blood pressure, reinforces the importance of promoting healthy dietary patterns in the university population.

Regarding sociodemographic factors, it was found that birthplace influences body composition, particularly among men, with significant differences in body fat percentage and hip circumference according to region of origin (Coastal vs. Highland). This highlights the role of geographic and cultural environments in shaping eating habits and body health.

In terms of parental background, although no statistically significant associations were found, a trend suggesting that paternal origin could slightly influence male students’ body composition was identified.

Overall, these findings emphasize the need to design specific nutritional intervention programs for university students, considering both individual and contextual factors. It is also crucial to integrate strategies that promote mindful eating and an active lifestyle to improve cardiovascular and metabolic health in this formative population.

Finally, it is recommended to incorporate health promotion policies in the university environment, addressing not only nutritional education but also academic stress management, recognizing the impact of these factors on quality of life and the prevention of chronic diseases from early adulthood.

## Data Availability

The raw data supporting the conclusions of this article will be made available by the authors, without undue reservation.
